# Determinants of Multidimensional and Physical Frailty and Their Individual Components: Interactions between Frailty Deficits

**DOI:** 10.3390/ijerph17228656

**Published:** 2020-11-21

**Authors:** Magdalena Sacha, Jerzy Sacha, Katarzyna Wieczorowska-Tobis

**Affiliations:** 1 Department of Palliative Medicine, Poznan University of Medical Sciences, 61-701 Poznan, Poland; tobis@ump.edu.pl; 2 Faculty of Physical Education and Physiotherapy, Opole University of Technology, 45-758 Opole, Poland; 3 Department of Cardiology, University Hospital, University of Opole, 45-401 Opole, Poland

**Keywords:** frailty, non-robust, FRAIL scale, Tilburg Frailty Indicator, determinants

## Abstract

*Purpose:* To identify the interrelations among determinants of multidimensional frailty, physical frailty, and their individual components. *Methods:* A group of 1024 community-dwelling people older than 65 years completed questionnaires regarding: multidimensional frailty (Tilburg Frailty Indicator, TFI) and physical frailty (FRAIL scale), and common frailty risk factors. *Results:* Multidimensional frailty was recognized in 559 subjects (54.6%) and determined by 13 factors (R^2^ = 0.21 in logistic regression). After incorporating TFI components to the models, the majority of previous risk factors became non-essential, and the frailty deficits mainly determined each other with R^2^ ranging between 0.07–0.67. Physical frailty and non-robust status (i.e., either physical frailty or pre-frailty) were recognized in 64 (6.3%) and 542 (52.9%) participants, and were determined by 5 factors (R^2^ = 0.33) and 11 factors (R^2^ = 0.34), respectively. Associations between the frailty deficits were detected within and between different dimensions (i.e., physical, psychological and social); the physical domain was mainly related to the psychological one which in turn was additionally associated with the social one. *Conclusion:* Frailty is the accumulation of deficits and is determined by factors other than the determinants of the individual deficits. The associations between deficits coming from various dimensions of human functioning presumably amplify their effects and accelerate frailty development.

## 1. Introduction

Frailty is a pre-clinical condition that is associated with a decline in physiological reserves among the elderly people and it predisposes them to various adverse outcomes including functional deterioration, disability and death [1,2,3]. Frailty is usually considered as a set of physical impairments, such as sarcopenia, weight loss, poor mobility and fatigue; but in fact, frailty is an accumulation of deficits in different dimensions of human functioning, i.e., physical, psychological and social ones; and for its diagnosis, a certain number of such deficits must be identified [3,4,5]. Many risk factors for frailty development have been determined; yet, frailty as an accumulation of impairments combines conditions which certainly constitute risk factors for each other and their interplay most likely amplify their effects. Moreover, since frailty is a multidimensional entity, the interaction between impairments in various dimensions presumably accelerates the overall functional degradation associated with aging [6,7].

To recognize physical and multidimensional frailty, respective diagnostic tools must be employed which should allow a quick detection of frailty symptoms and an early identification of subjects at risk. In terms of physical frailty, a questionnaire named the FRAIL scale appears to be a simple and sensitive measure for selecting people with physical impairments [8,9]; but in terms of multidimensional frailty, the Tilburg Frailty Indicator (TFI) is gaining popularity as an effective questionnaire for an early diagnosis of deficits in multiple dimensions [5]. Combination of unidimensional (i.e., physical) and multidimensional frailty diagnostic tools may yield more information about the character of functional disturbances associated with age than either of these tools employed exclusively. Indeed, it has been recently shown that a simultaneous employment of TFI and the FRAIL scale, may identify subgroups of the elderly people that present different functional profiles—i.e., those presenting predominantly social and psychological frailty or those with mainly physical deficits [7]. Such subgroups potentially require different management and, therefore, the approach to frail people should be individualized according to their functional state. However, for the individualized frailty prevention and treatment, determinants of frailty itself along with determinants of the individual frailty deficits should be recognized in order to design the appropriate strategy in a given deficits’ constellation. Moreover, particular attention should be paid to the interactions between frailty components originating from various domains, e.g., physical and psychological frailty deficits probably constitute a vicious cycle in which one feeds the development of the other [1,7]. An early recognition of subjects at risk is paramount to employing an effective preventative strategy against frailty; and, therefore, frailty screening and seeking its determinants should be focused on a general (not institutionalized) elderly population [7].

In this study, a large group of community-dwelling elderly people was investigated for the presence of frailty, its risk factors and the relationships between various deficits associated with aging. The primary goal of the study was to identify independent determinants of multidimensional and physical frailty, as well as, each of the frailty components in two diagnostic frailty tools, i.e., TFI (dedicated to multidimensional frailty) and the FRAIL scale (devoted to physical frailty) [5,8,9]. The secondary goal was to investigate the association and interaction between deficits in different frailty dimensions.

## 2. Materials and Methods

### 2.1. Participants

Community-dwelling people at the age of 65 years or older living in Opole District (southwest Poland) took part in this cross-sectional study. The participants were recruited during healthy lifestyle promotion meetings arranged by local community-based senior organizations between December 2017 and December 2018—in total, there were 30 meetings during this period, and they gathered around 50 participants on average. These meetings were devoted to all elderly people living in a region (not only to the organizations’ members) and they were advertised by suitable posters. There were no specific exclusion criteria except the age below 65 years and a lack of consent to take part in the study—due to these reasons, about one third of the meetings’ attendees were not eligible for this research. Since the study was conducted among people coming to the meetings, all participants were moving around by themselves and they were not dependent on other people, and therefore represented an active part of the elderly population. The subjects completed by themselves questionnaires concerning multidimensional and physical frailty, as well as risk factors related to frailty (selected on the basis of previous research on frailty) [5,8,9,10]. The questionnaires were anonymous and included a short description of the study rationale. The research protocol was approved by the Ethics Committee at the Poznan University of Medical Sciences and all participants gave their informed consent. More details on the activities of community-based senior organizations in Poland may be found elsewhere [11].

### 2.2. Frailty Instruments

Multidimensional frailty has been investigated by using part B of the TFI which consists of 15 frailty deficits arranged according to three different domains. The physical domain (0–8 points) contains eight items: poor physical health, unintentional weight loss, difficulty in walking, difficulty in maintaining balance, poor hearing, poor vision, lack of strength in hands, and physical tiredness. The psychological domain (0–4 points) consists of four components: problems with memory, feeling down, feeling nervous or anxious, and inability to cope with problems. The social domain (0–3 points) comprises three elements: living alone, missing other people, and lack of support from other people. The TFI total score may range from 0 to 15; by definition, frailty is recognized if the TFI score is at least 5 [5]. Part A of TFI contains risk factors leading to frailty which have been selected in the previous research on frailty, and this includes age, gender, education level, economic status, lifestyle, marital status, experiences with different unfavorable events in the recent period, and satisfaction with living conditions [5,10,12,13].

Physical frailty has been ascertained with the FRAIL scale which contains 5 components: physical tiredness/fatigue, inability to walk up one flight of stairs, inability to walk 200 m, unexplained body mass loss, and a number of chronic diseases [8,9]. Unexplained body mass loss is scored 1 if respondents communicate their weight loss of 6 kg or more during the last six months, or 3 kg or more during the last month. The presence of 5 or more chronic illnesses yields score 1, otherwise it is scored 0. FRAIL scale scores range from 0–5 and may reflect frail (3–5), prefrail (1–2), and robust (0) status [8,9].

The participants were also asked about a place of living (village or city), former occupation (physical or intellectual one) and if they are members of community-based senior organizations.

### 2.3. Statistical Analysis

The continuous variables were presented as mean ± standard deviation (SD). Categorical variables were presented as numeric values and percentages. Relationship between two variables was investigated with Pearson correlation. Independent determinants for different types of frailty and their components were identified with logistic regression through multiple testing—for each model, a determination coefficient was calculated which expressed the proportion of variance in the dependent variable explained by independent variables. Variables with *p* > 0.1 in adjusted analyses were not retained in the final model. To validate the models and exclude bias, a bootstrapping technique with 2000 samples was employed. In addition, the analyses (employing logistic regression) were performed to investigate the interaction between TFI components in determining another TFI component or FRAIL scale component. The associations between different frailty dimensions and their determinants were explored with multiple linear regression analysis—their interaction was checked with a calculation of centered product terms. Each model was validated in the bootstrapping analysis. The threshold probability of *p* < 0.05 was taken as the level of statistical significance. All analyses were performed using NCSS 12 Statistical Software (2018), NCSS, LLC, Kaysville, Utah, USA, and the Statistical Package for Social Sciences (SPSS, v. 22.0, IBM SPSS xStatistics, IBM Corporation, Chicago, IL, USA).

## 3. Results

### 3.1. Frailty Prevalence

Of the approximately 1500 attendees of the meetings arranged by senior organizations, 1024 community-dwelling individuals over the age of 65 years (72.6 ± 6.3 years; range 65–93 years; 270 males) took part in this cross-sectional study. The baseline participants’ characteristics are presented in Table 1. The multidimensional frailty was diagnosed in 559 subjects (54.6%), whereas physical frailty, pre-frailty and non-robust status (i.e., either physical frailty or pre-frailty) were recognized in 64 (6.3%), 478 (46.7%) and 542 (52.9%) participants, respectively.

### 3.2. Frailty Determinants

Numerous risk factors were independently associated with different types of frailty (Table 2), i.e., the multidimensional frailty was determined by 13 variables that explained 21% of the variance; whereas physical frailty and non-robust status were associated with 5 variables (explaining 33% of the variance) and 11 variables (explaining 34% of the variance), respectively. Age significantly increased the risk of multidimensional frailty akin to serious illness, the end of an important relationship, chronic diseases, and an inability to walk up one flight of stairs. However, male sex, a high school or university education level, a healthy lifestyle, participation in senior groups, living in a city or in a relationship as well as satisfaction with living conditions, they all reduced the likelihood of multidimensional frailty.

The risk of physical frailty was elevated by poor physical health, difficulty in walking or maintaining balance, and a lack of strength in hands. Of note, missing other people decreased the risk of being physically frail. The non-robust status was determined by more factors, i.e.,: serious illness, poor physical health, difficulty in walking or maintaining balance, poor vision, a lack of strength in hands, and feeling down increased the risk of being non-robust; whereas, a partially healthy or healthy lifestyle, participation in senior organizations, and serious illness of a loved person in the recent time independently diminished the risk.

The logistic regression analysis was performed to identify independent factors associated with each individual component of TFI and the FRAIL scale—the results are exhibited in Table 3. After incorporating TFI components to the models, majority of the previous risk factors of multidimensional frailty (Table 2) became non-essential, moreover, the TFI components appeared to be significantly related to each other. The regression models in Table 3 explain 7% to 67% (on average, 38%) of the variance of TFI deficits. In the validation bootstrapping analysis, all models in Table 2 and Table 3 appeared to be valid, and in general, only few variables (i.e., 5 out of 196) presented discordant significance compared to the primary models (Table 3). On average, each TFI element was independently associated with 4.9 different TFI elements and 3 other risk factors (Table 3). Specifically, physical tiredness, and living alone were related to 7 other TFI components; whereas, a lack of strength in hands, feeling down, and feeling nervous or anxious were associated with 6 different TFI items; difficulty in maintaining balance, poor vision, problems with memory, an inability to cope with problems, missing other people, and a lack of support from other people were related to 5 various TFI components—other components were associated with no more than 4 TFI items.

Regarding the FRAIL scale, after incorporating their components to the models, the variance of the particular scale components could be explained in 7% to 51% (on average, 33%), and they (in majority) revealed the association with one of the other FRAIL scale components (Table 3). In addition, some of them were related to feeling down and missing other people (Table 3), and there was a significant interaction between poor vision and feeling down in determining the non-robust status (Table 2).

### 3.3. Relationships between Frailty Dimensions

The associations between the TFI elements were detected not only within a given dimension (i.e., physical dimension, item 1–8; psychological dimension, item 9–12; and social dimension, item 13–15), but also between different dimensions. The scores for each dimension (i.e., the sums of corresponding deficits) significantly correlated with each other, i.e., the physical domain correlated with psychological and social ones (r = 0.43 and r = 0.15, respectively, *p* < 0.0001 for both), and the psychological domain correlated with social one (r = 0.27, *p* < 0.0001). However, in the multiple regression analysis, the physical domain was determined by the psychological domain only; the psychological domain was independently associated with both the physical and social ones; but the social domain was exclusively determined by the psychological one (Table 4). In the interaction analysis, there was no statistically significant buffering effect between the domains (Table 4). Table 5 presents determinants for each of the TFI domains pointing out which of the variables are independently associated with a given domain. The data in Table 4 and Table 5 reflect the associations among frailty deficits coming from different dimensions of human functioning, and in addition some interactions between individual deficits can be found (Table 5). Moreover, in Table 3, significant interactions are seen between physical and psychological TFI components, within some psychological TFI components, and between psychological and social ones.

## 4. Discussion

Multidimensional frailty has been recognized in 54.6% of the study population, whereas, physical frailty and pre-frailty have been diagnosed in 6.3% and 46.7%, respectively. This corresponds to the data coming from other populations and shows that the prevalence of these conditions is similar in different regions and cultures [14,15,16].

Numerous elements have been identified as independent risk factors for both multidimensional and physical frailty as well as non-robust status (i.e., either physical frailty or pre-frailty) [6,10,17,18]; however, for prevention or therapeutic intervention, such analyses may be incomplete since the individual frailty components could not be considered in these models and the associations between different frailty dimensions could not be discerned. In fact, frailty is an accumulation of deficits and as such is determined by factors other than factors determining its particular components. Therefore, for the purpose of intervention, determinants of the individual deficits should be considered along with determinants of their accumulation; moreover, the relationships between various dimensions of human functioning (i.e., physical, psychological and social) must be taken into account.

In line with TFI, the association among frailty deficits is especially valid between physical and psychological domains, i.e., most of the physical deficits were independently associated with psychological ones, and the correlation between these domains was quite high (i.e., r = 0.43). In a sample of more than 35,000 community-dwelling Dutch people older than 65 years, the correlation was very similar, i.e., equal to 0.45 [17]. Moreover, in the multivariate analysis, the physical domain of TFI was determined by only the psychological one, and there was no buffering effect of the social domain. Conversely, the psychological domain was impacted by the physical one, and in addition by the social one. There were also some interactions between physical and psychological TFI components in determining various frailty deficits. These observations indicate that physical frailty is associated with psychological frailty with a possible bidirectional causal relationship [1,19,20]. Indeed, there is a number of data showing that a poor cognitive performance predicts physical decline, but also, physical frailty may determine cognitive frailty which in turn may lead to dementia [21,22,23,24]. However, a precise mechanism how physical frailty or pre-frailty can cause a cognitive decline is not fully clarified [25,26,27].

Regarding the social domain in TFI, it had no independent effect on the TFI physical domain in the multivariate analysis. However, missing other people (i.e., a social component of TFI) significantly but inversely determined an inability to walk up one flight of stairs (i.e., a physical component of the FRAIL scale); moreover, it was also inversely associated with the prevalence of physical frailty. Hence, a lack of other people may probably impose some activities that in turn may result in higher physical tolerance. On the other hand, the association between social and psychological dimensions in TFI was significant and mutual in the regression models, and all psychological TFI deficits were determined by some social ones. Of note, living alone was inversely associated with problems with memory, and feeling nervous or anxious. This presumably stems from the fact that lonely people have to utilize their memory for daily needs, and they are not exposed to psychological tensions with home dwellers. There were also certain interactions between social and psychological TFI components in regard to some other frailty deficits.

The aforementioned inverse relationships between frailty components suggest that some deficits may prevent other ones. Indeed, circumstances and external stimuluses may provoke kinds of activity among the elderly subjects provided that the intensity of such factors does not cross the limits of their capabilities [11,28]. The necessity to address daily needs and different types of issues may access some energy layers in the elderly individuals and enable them to preserve good functioning and independence. Such mechanisms constitute fundamentals for the concept of aging in place, the ideology promoted worldwide by the World Health Organization (WHO) [29]. However, basic conditions for aging in place are some levels of competence and control over one’s environment [30,31,32]. Therefore, to ensure the wellbeing of elderly people in their place, a holistic approach is needed where the identification of factors predisposing them to and preventing them from different forms of frailty is paramount. In this context, a simultaneous employment of both unidimensional (i.e., physical) and multidimensional frailty diagnostic tools may more precisely characterize the deficits’ structure than any of these tools employed exclusively [7]. By uncovering the individual’s lacks and needs, one may more effectively assist in supporting his/her functional independence in aging in place.

From the practical point of view, the most crucial frailty risk factors are those which pose a chance of being modified by the appropriate management. Many of the common risk factors are not modifiable (e.g., age, sex, education level); yet, the majority of multidimensional and physical frailty components may be the subject of intervention; moreover, most of their determinants are in fact other frailty deficits suitable for modification. Among the physical TFI and FRAIL scale deficits, the majority reflect muscle weakness and sarcopenia which can be prevented and treated with the suitable training programs associated with proper nutritional interventions [33,34]. Problems with vision and hearing should be managed with appropriate glasses and hearing devices [35,36,37]; this is critical because, in our data, these sensory deficits are associated with the development of deficits in other frailty dimensions; in particular, they determine the memory problems. In fact, the physical impairments are mutually associated with the psychological ones, and the most influential ingredient of this relationship is ‘feeling down’. Indeed, depression is a common element in aging processes, and thus, it must be early recognized and properly treated in order to improve people’s mood and their motivation for an active life [38,39,40]. In this context, our study suggests that social deficits may intensify psychological problems, due to their mutual association. Loneliness and lack of support from other people are the principal reasons for low quality of life in elderly people, and they contribute to functional deterioration and mortality [18,41,42,43,44]. Therefore, proper social programs involving families and local communities should be arranged in order to alleviate loneliness associated with aging.

The awareness of the interplay among deficits is paramount in designing individualized management in the elderly people. Ideally, a profile of functional abnormalities in a given subject should be first recognized, and then, after considering the deficits’ relationships, an individualized interventional strategy could be appropriately designed. The findings of the present study are in line with the recent study where simultaneous employment of TFI and the FRAIL scale enabled us to identify subgroups of elderly people presenting different functional profiles, i.e., those presenting predominantly social and psychological frailty and those with mainly physical deficits [7]. Such different subgroups probably require different management and, therefore, the approach to frail subjects should be individualized according to the functional state. However, the feasibility, practicability and clinical efficiency of such a strategy must be first prospectively tested in subjects with different degrees of functional deterioration, before it will be recommended for a wide application.

The present study has some limitations that need to be acknowledged. The observational and cross-sectional nature of this research does not allow cause–effect interpretations of the associations between frailty deficits and various risk factors. Despite the internal validation with the bootstrapping technique, the study results should be externally validated in other seniors’ groups. The use of self-reported questionnaires distributed among the elderly people attending healthy lifestyle promotion meetings may impose some selection bias. The variance of some deficits could only be explained in a small portion (e.g., a lack of support from other people), which means that factors other than those considered in this study determine these deficits and, therefore, it requires further investigation. Both TFI and the FRAIL scale have been validated in different populations and clinical circumstances, however, their value in picturing different functional profiles of elderly people and designing the individualized interventional strategies have never been tested. The strength of this study is the observation that the individual frailty deficits determine each other, and the impairments in the physical domain are mainly affected by psychological deficits which in turn are additionally impacted by social deficits. This may shed more light for mechanisms accelerating frailty development and help to design a more comprehensive approach to frailty [6,7,11,17,18].

## 5. Conclusions

Multidimensional frailty and non-robust physical status (i.e., either physical frailty or prefrailty) are common in community-dwelling elderly people, and numerous demographic and clinical variables are associated with these conditions. However, frailty as an accumulation of deficits is determined by factors other than factors determining its individual deficits, and the interplay between these deficits presumably amplify their effects and may accelerate frailty development. In particular, a bidirectional association exists between physical and psychological frailty dimensions; the latter is additionally impacted by deficits in the social domain. Therefore, for preventative and therapeutic purposes, determinants of each individual deficit should be considered along with determinants of their accumulation; and the associations between various dimensions of human functioning should also be taken into account. By unravelling a functional profile in a given elderly subject, an individualized management may be designed, however, the feasibility and clinical efficacy of such an approach need to be tested in suitable prospective studies.

## Figures and Tables

**Table 1:** Study group characteristics.

Characteristic	Overall Group
Age (years)	72.6 ± 6.3
Male sex	270 (26.4)
Primary school education level	258 (25.2)
High school education level	464 (45.3)
University education level	302 (29.5)
Low economic status	152 (14.8)
Moderate economic status	835 (81.5)
High economic status	37 (3.6)
Unhealthy lifestyle	54 (5.3)
Partially healthy lifestyle	532 (52.0)
Healthy lifestyle	438 (42.8)
Participation in a senior organization	460 (44.9)
Living in a city	746 (72.9)
Living in a relationship	529 (51.7)
Former intellectual occupation	646 (63.1)
Death of a loved person in the recent time	389 (38.0)
Serious illness in the recent time	229 (22.4)
Serious illness of a loved person in the recent time	245 (23.9)
End of an important relationship in the recent time	70 (6.8)
Traffic accident in the recent time	59 (5.8)
Criminal event in the recent time	23 (2.2)
Satisfaction with living conditions	903 (88.2)
Number of chronic diseases *	1.9 ± 1.6
Inability to walk up one flight of stairs *	87 (8.5)
Inability to walk 200 m *	101 (9.9)
Physical Domain of Tilburg Frailty Indicator (TFI)	
Poor physical health	331 (32.3)
2. Unexplained body mass loss *	133 (13.0)
3. Difficulty in walking	371 (36.2)
4. Difficulty in maintaining balance	261 (25.5)
5. Poor hearing	358 (35.0)
6. Poor vision	414 (40.4)
7. Lack of strength in hands	283 (27.6)
8. Physical tiredness/fatigue *	465 (45.4)
Psychological Domain of TFI	
9. Problems with memory	138 (13.5)
10. Feeling down	671 (65.5)
11. Feeling nervous or anxious	675 (65.9)
12. Inability to cope with problems	188 (18.4)
Social Domain of TFI	
13. Living alone	384 (37.5)
14. Missing other people	682 (66.6)
15. Lack of support from other people	185 (18.1)
Sum of physical deficits (components: 1–8)	2.6 ± 2.1
Sum of psychological deficits (components: 9–12)	1.6 ± 1.1
Sum of social deficits (components: 13–15)	1.2 ± 0.9
Total score of TFI (all components)	5.4 ± 3.1
Multidimensional frailty according to TFI	559 (54.6)
Total score for physical frailty according to FRAIL scale	0.8 ± 0.9
Physical frailty according to the FRAIL scale	64 (6.3)
Physical pre-frailty according to the FRAIL scale	478 (46.7)
Non-robust status according to the FRAIL scale	542 (52.9)

Notes: Values are mean ± SD or n (%). * Denotes components of the FRAIL scale.

**Table 2:** Independent risk factors of multidimensional and physical frailty as well as non-robust status.

Independent Variables	Multidimensional Frailty R^2^ = 0.21, *p* < 0.00001		Physical Frailty R^2^ = 0.33, *p* < 0.00001		Non-Robust Status R^2^ = 0.34, *p* < 0.00001	
	B (SE)	*p*-Value	B (SE)	*p*-Value	B (SE)	*p*-Value
Age	**0.07 (0.01)**	**<0.00001**				
Male sex	**−** **0.38 (0.17)**	**<0.05**				
High school education level	**−** **0.69 (0.2)**	**<0.001**			−0.42 (0.21)	0.051
University education level	**−** **0.66 (0.22)**	**<0.01**			−0.41 (0.23)	0.08
Moderate economic status						
High economic status						
Partially healthy lifestyle	−0.68 (0.37)	0.07			**−** **1.43 (0.48)**	**<0.01**
Healthy lifestyle	**−** **1.05 (0.38)**	**<0.01**			**−** **1.64 (0.48)**	**<0.001**
Participation in a senior organization	**−** **0.38 (0.15)**	**<0.05**			**−** **0.45 (0.16)**	**<0.01**
Living in a city	**−** **0.47 (0.18)**	**<0.01**			−0.34 (0.19)	0.07
Living in a relationship	**−** **0.45 (0.16)**	**<0.01**				
Former intellectual occupation						
Death of a loved person in the recent time					0.31 (0.16)	0.06
Serious illness in the recent time	**0.69 (19)**	**<0.001**			**0.48 (0.2)**	**<0.05**
Serious illness of a loved person in the recent time	0.3 (0.17)	0.08			**−** **0.39 (0.19)**	**<0.05**
End of an important relationship in the recent time	**0.87 (0.33)**	**<0.01**				
Traffic accident in the recent time	0.61 (0.33)	0.06				
Criminal event in the recent time						
Satisfaction with living conditions	**−** **2.12 (0.37)**	**<0.00001**				
Number of chronic diseases *	**0.12 (0.05)**	**<0.05**	–	–	–	–
Inability to walk up one flight of stairs *	**0.96 (0.34)**	**<0.01**	–	–	–	–
Inability to walk 200 m *			–	–	–	–
Physical Domain of Tilburg Frailty Indicator (TFI)						
Poor physical health	–	–	**2.08 (0.44)**	**<0.00001**	**1.18 (0.2)**	**<0.00001**
2. Unexplained body mass loss *	–	–	–	–	–	–
3. Difficulty in walking	–	–	**0.97 (0.42)**	**<0.05**	**0.85 (0.18)**	**<0.00001**
4. Difficulty in maintaining balance	–	–	**1.48 (0.36)**	**<0.0001**	**0.65 (0.22)**	**<0.01**
5. Poor hearing	–	–				
6. Poor vision	–	–			**0.53 (0.17) a**	**<0.01**
7. Lack of strength in hands	–	–	**0.75 (0.32)**	**<0.05**	**1.13 (0.21)**	**<0.00001**
8. Physical tiredness/fatigue *	–	–	–	–	–	–
Psychological Domain of TFI						
9. Problems with memory	–	–				
10. Feeling down	–	–			**0.53 (0.17) a**	**<0.01**
11. Feeling nervous or anxious	–	–				
12. Inability to cope with problems	–	–			−0.44 (0.24)	0.07
Social Domain of TFI						
13. Living alone	–	–				
14. Missing other people	–	–	**−0.72 (0.31)**	**<0.05**		
15. Lack of support from other people	–	–				

Notes: * Denotes components of the FRAIL scale. Same letters (i.e., a) next to the coefficients correspond to the variables which significantly interact with *p*-values less than 0.05. Statistically significant coefficients and *p*-values are marked in bold. The validation bootstrapping analysis confirmed the statistical significance of the models and their variables.

**Table 3:** Independent determinants for each component of TFI and the FRAIL scale.

Independent Variables	1. Poor Physical Health R^2^ = 0.49, *p* < 0.00001		2. Unexplained Body Mass Loss * R^2^ = 0.07, *p* < 0.00001		3. Difficulty in Walking R^2^ = 0.4, *p* < 0.00001		4. Difficulty in Maintaining Balance R^2^ = 0.33, *p* < 0.00001		5. Poor Hearing R^2^ = 0.23, *p* < 0.00001		6. Poor Vision R^2^ = 0.48, *p* < 0.00001	
	B (SE)	*p*-Value	B (SE)	*p*-Value	B (SE)	*p*-Value	B (SE)	*p*-Value	B (SE)	*p*-Value	B (SE)	*p*-Value
Age			**0.03 (0.01)**	**<0.05**	**0.06 (0.01)**	**<0.00001**	**0.04 (0.01)**	**<0.05**	**0.05 (0.01)**	**<0.0001**		
Male sex									**0.47 (0.16)**	**<0.01**		
High school education level											**−0.55 (0.18)**	**<0.01**
University education level											**−0.49 (0.19)**	**<0.05**
Moderate economic status												
High economic status												
Partially healthy lifestyle												
Healthy lifestyle												
Participation in a senior group									**−0.56 (0.15)**	**<0.001**		
Living in a city												
Living in a relationship							**−0.63 (0.18)**	**<0.001**	−0.29 (0.15)	0.054 ^‡^		
Former intellectual occupation												
Death of a loved person in the recent time												
Serious illness in the recent time												
Serious illness of a loved person in the recent time												
End of an important relationship in the recent time												
Traffic accident in the recent time	**−1.11 (0.39)**	**<0.01**			**0.66 (0.33)**	**<0.05 ^†^**						
Criminal event in the recent time	**1.95 (0.56)**	**<0.001**										
Satisfaction with living conditions	**−1.38 (0.27)**	**<0.00001**	**−0.91 (0.24)**	**<0.001**								
Number of chronic diseases *	**0.25 (0.05)**	**<0.00001**										
Inability to walk up one flight of stairs *	**0.99 (0.31)**	**<0.01**	0.55 (0.28)	0.053 ^‡^								
Inability to walk 200 m *					**1.08 (0.31)**	**<0.001**	**0.94 (0.27)**	**<0.001**				
Physical Domain of Tilburg Frailty Indicator (TFI)												
Poor physical health	–	–			**1.01 (0.18)**	**<0.00001**	**0.58 (0.19)**	**<0.01**				
2. Unexplained body mass loss *			–	–								
3. Difficulty in walking	**1.02 (0.18)**	**<0.00001**			–	–	**1.18 (0.19) ^a^**	**<0.00001**			0.29 (0.16)	0.06 ^a^
4. Difficulty in maintaining balance	**0.58 (0.2)**	**<0.01**			**1.12 (0.19)**	**<0.00001**	–	–				
5. Poor hearing									–	–	**0.84 (0.15)**	**<0.00001**
6. Poor vision									**0.91 (0.14)**	**<0.00001**	–	–
7. Lack of strength in hands					**0.6 (0.19)**	**<0.01**	**0.67 (0.19)**	**<0.001**			**0.46 (0.17)**	**<0.01**
8. Physical tiredness/fatigue *	**1.25 (0.18)**	**<0.00001**	**0.66 (0.21)**	**<0.01**	**0.92 (0.17)**	**<0.00001**	**0.6 (0.2) ^b^**	**<0.01**			**0.63 (0.16)**	**<0.0001**
Psychological Domain of TFI												
9. Problems with memory							**1.19 (0.23) ^ab^**	**<0.00001**	**0.77 (0.21)**	**<0.001**	**0.68 (0.22)**	**<0.01 ^a^**
10. Feeling down												
11. Feeling nervous or anxious											**0.4 (0.15)**	**<0.01**
12. Inability to cope with problems	**0.92 (0.22)**	**<0.0001**										
Social Domain of TFI												
13. Living alone			−0.35 (0.21)	0.09	−0.3 (0.17)	0.08						
14. Missing other people												
15. Lack of support from other people	0.39 (0.21)	0.07										
**Part II**												
**Independent Variables**	**7. Lack of Strength in Hands** **R^2^ = 0.45, *p* < 0.00001**		8. Physical Tiredness * **R^2^ = 0.51, *p* < 0.00001**		**9. Problems with Memory** **R^2^ = 0.23, *p* < 0.00001**		**10. Feeling Down** **R^2^ = 0.57, *p* < 0.00001**		**11. Feeling Nervous or Anxious** **R^2^ = 0.44, *p* < 0.00001**		**12. Inability to Cope with Problems** **R^2^ = 0.27, *p* < 0.00001**	
	**B (SE)**	***p*-value**	**B (SE)**	***p*-value**	**B (SE)**	***p*-value**	**B (SE)**	***p*-value**	**B (SE)**	***p*-value**	**B (SE)**	***p*-value**
Age					**0.05 (0.02)**	**<0.01**						
Male sex	**−0.7 (0.2)**	**<0.001**										
High school education level											**−0.44 (0.22)**	**<0.05 ^†^**
University education level												
Moderate economic status			**0.52 (0.24)**	**<0.05**	**−0.61 (0.25)**	**<0.05**						
High economic status			**0.97 (0.47)**	**<0.05**	**−1.39 (0.69)**	**<0.05**						
Partially healthy lifestyle			**−1.25 (0.42)**	**<0.01**								
Healthy lifestyle			**−1.41 (0.43)**	**<0.001**								
Participation in a senior group												
Living in a city			**−0.71 (0.18)**	**<0.0001**								
Living in a relationship							0.35 (0.21)	0.095	−0.39 (0.21)	0.069		
Former intellectual occupation									**0.54 (0.16)**	**<0.001**		
Death of a loved person in the recent time									**−0.34 (0.16)**	**<0.05**		
Serious illness in the recent time					**0.46 (0.23)**	**<0.05**					**0.56 (0.2)**	**<0.01**
Serious illness of a loved person in the recent time												
End of an important relationship in the recent time												
Traffic accident in the recent time	**0.97 (0.32)**	**<0.01**										
Criminal event in the recent time												
Satisfaction with living conditions							**−1.06 (0.36)**	**<0.01**			**−1.01 (0.24)**	**<0.0001**
Number of chronic diseases *												
Inability to walk up one flight of stairs *											**0.6 (0.29)**	**<0.05**
Inability to walk 200 m *												
Physical Domain of Tilburg Frailty Indicator (TFI)												
Poor physical health			**1.13 (0.18)**	**<0.00001**							**0.82 (0.2)**	**<0.0001**
2. Unexplained body mass loss *			**0.53 (0.25)**	**<0.05**								
3. Difficulty in walking	**0.73 (0.18)**	**<0.0001**	**0.92 (0.18)**	**<0.00001**								
4. Difficulty in maintaining balance	**0.73 (0.19) ^a^**	**<0.001**	**0.58 (0.2)**	**<0.01**	**1.19 (0.22)**	**<0.00001**						
5. Poor hearing					**0.74 (0.22)**	**<0.001**						
6. Poor vision	**0.53 (0.17)**	**<0.01**	**0.7 (0.16)**	**<0.0001**	**0.67 (0.22)**	**<0.01**						
7. Lack of strength in hands	–	–	**1.21 (0.19)**	**<0.00001**			**0.57 (0.21) ^a^**	**<0.01**	**0.5 (0.19)**	**<0.01**		
8. Physical tiredness/fatigue *	**1.26 (0.19)**	**<0.00001**	–	–			**0.64 (0.17)**	**<0.001**				
Psychological Domain of TFI												
9. Problems with memory					–	–					**0.8 (0.23)**	**<0.001**
10. Feeling down	**0.61 (0.21) ^ab^**	**<0.01**	**0.56 (0.17)**	**<0.001**			–	–	**1.53 (0.16) ^a^**	**<0.00001**	**0.78 (0.27)**	**<0.01**
11. Feeling nervous or anxious	0.34 (0.2) ^b^	0.097					**1.48 (0.16) ^ab^**	**<0.00001**	–	–	**0.8 (0.25)**	**<0.01**
12. Inability to cope with problems	**0.44 (0.21)**	**<0.05**			**0.83 (0.23) ^a^**	**<0.001**	**0.93 (0.26)**	**<0.001**	**0.77 (0.24)**	**<0.01**	–	–
Social Domain of TFI												
13. Living alone					**−0.47 (0.22) ^a^**	**<0.05**	**0.65 (0.22) ^b^**	**<0.01**	**−0.63 (0.22)**	**<0.01**		
14. Missing other people							**0.63 (0.16)**	**<0.0001**	**0.75 (0.16) ^a^**	**<0.00001**	**0.47 (0.23)**	**<0.05**
15. Lack of support from other people									**0.75 (0.23)**	**<0.01**		
**Part III**												
**Independent Variables**	**13. Living Alone** **R^2^ = 0.64, *p* < 0.00001**		**14. Missing Other People** **R^2^ = 0.41, *p* < 0.00001**		**15. Lack of Support from Other People** **R^2^ = 0.12, *p* < 0.00001**		Inability to Walk Up One Flight of Stairs * **R^2^ = 0.44, *p* < 0.00001**		Inability to Walk 200 m * **R^2^ = 0.46, *p* < 0.00001**		More than Four Illnesses * **R^2^ = 0.15, *p* < 0.00001**	
	**B (SE)**	***p*-value**	**B (SE)**	***p*-value**	**B (SE)**	***p*-value**	**B (SE)**	***p*-value**	**B (SE)**	***p*-value**	**B (SE)**	***p*-value**
Age					**−0.06 (0.2)**	**<0.001**					**0.05 (0.15)**	**0.001**
Male sex							−0.64 (0.37)	0.09				
High school education level			−0.37 (0.2)	0.054					−0.56 (0.33)	0.08	**0.57 (0.25)**	**<0.05**
University education level			**−0.47 (0.21)**	**<0.05**					**−1.95 (0.47)**	**<0.0001**	**0.74 (0.28)**	**<0.01**
Moderate economic status					**−0.67 (0.23)**	**<0.01**						
High economic status												
Partially healthy lifestyle												
Healthy lifestyle			**−0.35 (0.14)**	**<0.05**								
Participation in a senior group			0.27 (0.15)	0.07					**−1.01 (0.36)**	**<0.01**	−0.38 (0.21)	0.08
Living in a city	**0.68 (0.21)**	**<0.01**	**−0.55 (0.18)**	**<0.01**			−0.61 (0.33)	0.06	**0.91 (0.34)**	**<0.01**		
Living in a relationship	**−3.93 (0.24)**	**<0.00001**										
Former intellectual occupation												
Death of a loved person in the recent time	**0.45 (0.19)**	**<0.05**					**0.82 (0.32)**	**<0.05**				
Serious illness in the recent time											**1.16 (0.21)**	**<0.0001**
Serious illness of a loved person in the recent time							**−1.1 (0.42)**	**<0.01**				
End of an important relationship in the recent time	0.66 (0.37)	0.07							**0.94 (0.46)**	**<0.05 †**		
Traffic accident in the recent time											**0.82 (0.34)**	**<0.05**
Criminal event in the recent time												
Satisfaction with living conditions					−0.47 (0.26)	0.07 ^‡^			**−1.21 (0.35)**	**<0.001**		
Number of chronic diseases *							**0.25 (0.08)**	**<0.01**			–	–
Inability to walk up one flight of stairs *			**−0.66 (0.26)**	**<0.05**			–	–	**3.66 (0.34)**	**<0.00001**		
Inability to walk 200 m *							**3.7 (0.32)**	**<0.00001**	–	–		
Physical Domain of Tilburg Frailty Indicator (TFI)												
Poor physical health					**0.49 (0.2)**	**<0.05**	**1.26 (0.32)**	**<0.0001**			**0.77 (0.21)**	**<0.001**
2. Unexplained body mass loss *	**−0.62 (0.29)**	**<0.05**										
3. Difficulty in walking									**1.42 (0.32)**	**<0.0001**		
4. Difficulty in maintaining balance												
5. Poor hearing	**−0.44 (0.2)**	**<0.05**			**0.42 (0.18)**	**<0.05**						
6. Poor vision												
7. Lack of strength in hands												
8. Physical tiredness/fatigue *											**0.47 (0.22)**	**<0.05**
Psychological Domain of TFI												
9. Problems with memory	**−0.74 (0.28)**	**<0.01**										
10. Feeling down	**0.59 (0.22)**	**<0.01**	**0.62 (0.16) ^a^**	**<0.0001**								
11. Feeling nervous or anxious	**−0.58 (0.22)**	**<0.01**	**0.7 (0.16) ^a^**	**<0.0001**	**0.71 (0.22)**	**<0.01**					**−0.44 (0.21)**	**<0.05**
12. Inability to cope with problems			**0.45 (0.22) ^b^**	**<0.05**	0.41 (0.22)	0.06						
Social Domain of TFI												
13. Living alone	–	–	**0.72 (0.16)**	**<0.00001**	**0.7 (0.18)**	**<0.001**						
14. Missing other people	**0.86 (0.22)**	**<0.0001**	–	–	**0.5 (0.22)**	**<0.05**	**−0.78 (0.32)**	**<0.05**				
15. Lack of support from other people	**0.78 (0.25)**	**<0.01**	**0.49 (0.22) ^b^**	**<0.05**	–	–			−0.63 (0.38)	0.098		

Notes: * Denotes components of the FRAIL scale. ^†^ Denotes variables that did not reach significance in the validation bootstrapping analysis. ^‡^ Denotes variables that reached significance in the validation bootstrapping analysis, despite they were not significant in the primary model. Same letters (i.e., ^a^, ^b^) next to the coefficients correspond to the variables which significantly interact with *p*-values less than 0.05. Since the number of subjects with more than five illnesses was low, the dataset had quasi-complete separation, therefore “more than four illnesses” criterion was used in the regression analysis. Statistically significant coefficients and *p*-values are marked in bold.

**Table 4:** The association between different frailty domains according to TFI and their interactions in the multiple regression analysis.

Independent Variables	TFI Physical Domain R^2^ = 0.19, *p* < 0.00001		*p*-Value for Interaction
	B (SE)	*p*-Value	
TFI psychological domain	**0.82 (0.06)**	**<0.001**	0.06
TFI social domain	0.08 (0.07)	0.26	
	**TFI Psychological Domain** **R^2^ = 0.22, *p* < 0.00001**		
TFI physical domain	**0.2 (0.01)**	**<0.001**	0.082
TFI social domain	**0.25 (0.03)**	**<0.001**	
	**TFI Social Domain** **R^2^ = 0.07, *p* < 0.00001**		
TFI physical domain	0.02 (0.01)	0.15	0.43
TFI psychological domain	**0.2 (0.03)**	**<0.001**	

Notes: Statistically significant coefficients and *p*-values are marked in bold. The validation bootstrapping analysis confirmed the statistical significance of the models and their variables.

**Table 5:** Independent determinants for each TFI domain.

Independent Variables	TFI Physical Domain R^2^ = 0.45, *p* < 0.00001		TFI Psychological Domain R^2^ = 0.28, *p* < 0.00001		TFI Social Domain R^2^ = 0.31, *p* < 0.00001	
	B (SE)	*p*-Value	B (SE)	*p*-Value	B (SE)	*p*-Value
Age	**0.07 (0.01)**	**<0.00001**				
Male sex	**−0.27 (0.12)**	**<0.05**				
University/high school/primary education level						
High/moderate/low economic status					**−0.14 (0.06)**	**<0.05**
Healthy/partially healthy/unhealthy lifestyle	**−0.35 (0.09)**	**<0.001**			**−0.1 (0.4)**	**<0.05**
Participation in a senior group	**−0.63 (0.11)**	**<0.00001**				
Living in a city	**−0.29 (0.12)**	**<0.05**				
Living in a relationship					**−0.74 (0.05)**	**<0.00001**
Former intellectual occupation	**−0.38 (0.15)**	**<0.05**				
Death of a loved person in the recent time			**−0.16 (0.07)**	**<0.05**	**0.13 (0.5)**	**<0.05**
Serious illness in the recent time	**0.29 (0.13)**	**<0.05**	**0.3 (0.08)**	**<0.001**		
Serious illness of a loved person in the recent time					**−0.1 (0.06)**	**<0.05**
End of an important relationship in the recent time					**0.28 (0.1)**	**<0.01**
Traffic accident in the recent time	**0.46 (0.22)**	**<0.05**				
Criminal event in the recent time						
Satisfaction with living conditions	**−0.69 (0.18)**	**<0.0001**	**−0.48 (0.1)**	**<0.00001**		
Number of chronic diseases *	**0.14 (0.03)**	**<0.0001**				
Inability to walk up one flight of stairs *	**0.73 (0.23)**	**<0.01**				
Inability to walk 200 m *	**0.77 (0.22)**	**<0.001**				
Physical Domain of Tilburg Frailty Indicator (TFI)						
Poor physical health	–	–	**0.23 (0.08)**	**<0.01**		
2. Unexplained body mass loss *	–	–	**0.2 (0.09)**	**<0.05**		
3. Difficulty in walking	–	–				
4. Difficulty in maintaining balance	–	–	**0.18 (0.08)**	**<0.05**		
5. Poor hearing	–	–				
6. Poor vision	–	–	**0.25 (0.07)**	**<0.001**		
7. Lack of strength in hands	–	–	**0.29 (0.08)**	**<0.001**		
8. Physical tiredness/fatigue *	–	–	**0.15 (0.07)**	**<0.05**		
Psychological Domain of TFI						
9. Problems with memory	**0.88 (0.16) ^ab^**	**<0.00001**	–	–		
10. Feeling down	**0.52 (0.12) ^ac^**	**<0.0001**	–	–	**0.25 (0.06)**	**<0.0001**
11. Feeling nervous or anxious			–	–	**0.18 (0.05)**	**<0.01**
12. Inability to cope with problems	**0.53 (0.14)**	**<0.001**	–	–	**0.17 (0.07)**	**<0.05**
Social Domain of TFI						
13. Living alone					–	–
14. Missing other people			**0.44 (0.06)**	**<0.00001**	–	–
15. Lack of support from other people	**0.4 (0.14) ^bc^**	**<0.01**	**0.29 (0.08)**	**<0.001**	–	–

Notes: * Denotes components of the FRAIL scale. Same letters (i.e., ^a^, ^b^, ^c^) next to the coefficients correspond to the variables which significantly interact with *p*-values less than 0.05. Of note, the interaction was inverse between ‘problems with memory’ and ‘lack of support from other people’; other interactions were direct. Statistically significant coefficients and *p*-values are marked in bold. The validation bootstrapping analysis confirmed the statistical significance of the models and their variables.
